# Supplementary material for: Are carbon emissions trading and green financial instruments synergistic? -Comprehensive quantitative research based on content analysis
Source: PLoS One. 2024 Mar 7;19(3):e0298601. doi: 10.1371/journal.pone.0298601 (PMC10919601; doi:10.1371/journal.pone.0298601)
Supplement: S1 Data — (ZIP) [file pone.0298601.s001.zip › Data/地级市Z_F工作报告.zip!/─■╧─/╥°┤¿╩╨/└┤╫╘╣1⁄2╓┌║┼í╛┬φ┐╦╩2╛▌═°í┐_2013.docx]

各位代表：

现在，我代表市人民政府向大会作政府工作报告，请予审议，并请市政协委员和其他列席会议的同志提出意见。

一、过去五年工作回顾

2008年以来的五年，是银川发展历程中不平凡、不寻常的五年。在自治区党委、政府和市委的正确领导下，在市人大、市政协的监督支持下，市政府团结带领全市各族人民，认真贯彻落实科学发展观，抢抓西部大开发和沿黄经济区建设等重大战略机遇，积极应对国际金融危机等各种困难和挑战，坚持稳中求进、稳中求快和经济工作项目化，全力以赴保增长、调结构、转方式、促改革、惠民生，奋力建设“两个最适宜”城市，圆满完成了市十三届人大一次会议确定的各项目标任务，实现了经济持续快速发展和社会和谐稳定，书写了科学发展、跨越发展的崭新篇章。

——综合实力显著提升。主要经济指标连续五年保持两位数增长。今年地区生产总值突破千亿元大关，达到1140.83亿元，是2007年的2.6倍，年均增长13.1%；完成规模以上工业增加值430亿元，是2007年的2.7倍，年均增长17％；地方公共财政预算收入突破百亿元大关，达到113.13亿元，是2007年的4倍，年均增长33.4%；完成全社会固定资产投资918.73亿元, 是2007年的3.1倍，五年累计达到3159亿元，年均增长26.9%；完成社会消费品零售总额316.02亿元，是2007年的2.5倍，年均增长18.9%；城镇居民人均可支配收入和农民人均纯收入分别达到21900元和8068元，是2007年的1.8倍和1.9倍，年均增长12.5%和13.4%。县域经济跨越发展，灵武市跻身全国科学发展百强县（市），贺兰、永宁两县在“西部百强县”中争先进位。“五创”目标全面实现，“新五创”旗开得胜，获得全国文明城市、全国创业先进城市等20余项殊荣，城市综合竞争力明显提升。

——发展方式加快转变。深入实施“兴工强市”战略，力促优势特色产业做大做强。工业“一强四优五新”产业支撑作用凸显，宁东能源化工基地、银川经济技术开发区等园区建设不断提速，灵武羊绒工业园升级为国家级高新技术产业开发区，银川科技园、滨河新区工业园启动建设；65户工业企业位列全区百强企业，培育162户“小巨人企业”，工业发展质量和效益稳步提高。农业“两强四优四新”产业规模不断扩大，粮食生产实现“九连增”，设施园艺达到33万亩，鲜奶、水产总产均比2007年增长20%以上，有机水稻种植面积达到11.2万亩；贺兰山东麓葡萄文化产业带建设迅速起步，轩尼诗等世界著名品牌企业纷纷入驻。服务业“三个中心一个目的地”格局初步形成，空港、陆港、银川物流港建设全面推进，商贸业态提档升级；总部经济、星级酒店、会展业加快发展，动漫、创意等新兴产业初步发展，进入全国服务业综合改革试点城市、流通领域现代物流示范城市和电子商务示范城市行列。运动休闲城市品牌逐步形成，旅游接待人数、旅游收入年均分别增长23.3%和36.4%。科技进步对经济增长的贡献率达到49%，成为国家创新型试点城市。节能减排成效明显，万元GDP综合能耗累计下降24.1%，主要污染物减排任务全面完成，灵武再生资源循环经济示范区成为国家“城市矿产”示范基地。

——城乡面貌变化巨大。完成《银川市城市总体规划（2010-2020）》修编，启动滨河新区规划建设，加快建设阅海湾中央商务区，城市发展空间不断拓展，建成区面积由107平方公里扩大至135.1平方公里，城市化率达到73.2%，比2007年提高10个百分点。持续实施以道路、水系、绿化和特色街区改造为重点的城乡环境综合整治，大力推进以“洁净银川”、亮化美化为重点的“六大工程”。“三馆两中心”、览山公园、新火车站、贺兰山体育场等一批地标式建筑相继建成；新建扩建沈阳路、长城路等城市道路100余条，改造特色街区、小街巷175条，快速公交一号线建成运营；完成老旧小区和城市棚户区改造1000万平方米，城市供水、供暖、污水处理等基础设施体系逐步完善；市区东南、北部水系全线贯通，新增湖泊湿地1250公顷，建成区绿化覆盖率达到42.87%，森林覆盖率达到14.4%，建成海宝公园、唐徕公园市区段，完成红花渠改造，城市空气质量优良天数连续五年位居西北地区省会城市首位；统筹城乡发展有力推进，掌政等小城镇建设初具规模，新建“塞上农民新居”示范点69个，改造农村危房20880户，完成农村环境集中连片整治296个，农村面貌明显改观。“塞上湖城、西夏古都”城市特色更加显现，中心城市带动和沿黄城市带龙头作用进一步增强。

——民计民生持续改善。每年为民办好10件实事，并在人代会上向代表报告完成情况，公共财政预算收入优先用于改善民生。累计投资34.7亿元，新建改造中小学、幼儿园300余所，建成市职教中心，全面实现“两基”和基本普及高中阶段教育目标。完成市属5家医院、4所社区卫生服务中心等一批新建改造项目，建成标准化村卫生室69所，在全国率先实施人人享有基本医疗卫生服务试点，基本药物制度实现全覆盖，医疗服务水平和公共卫生保障能力不断提升。率先提出创建“食品安全城市”，食品药品监管卓有成效。文化惠民工程扎实推进，“湖城之夏·广场文化季”等群众性文化活动蓬勃开展，大型舞剧《月上贺兰》荣获多项国家大奖，成功承办第22届全国图书交易博览会，农家书屋实现全覆盖。银川汽车摩托车旅游节、贺兰山岩画艺术节、龙舟节等品牌活动影响日益广泛。“全民创业”深入开展，覆盖城乡的创业就业服务体系初步建立，累计新增城镇就业25万人。社会保障、社会救助体系逐步完善，率先在全区实现城乡居民养老保险全覆盖，在全国首创将城镇“三无人员”等弱势群体集中供养，建成社区老年活动中心60个。住房保障政策、制度不断健全完善，累计建设各类保障性住房281万平方米。扶贫开发、生态移民扎实推进，建成生态移民安置区7个、移民20139人。着力稳定居民生活必需品价格，建设社区平价蔬菜直销店，及时出台蔬菜、牛羊肉价格临时干预措施，保障了中低收入群众基本生活。加强和创新社会管理，“平安银川”建设深入推进，治安突出问题有效治理，安全生产控制指标连续五年下降，防灾减灾、应急管理体系逐步健全。民族团结进步创建活动扎实开展，人民防空、国防动员和后备力量建设稳步推进，荣获全国双拥模范城“七连冠”。

——发展活力日益增强。开通银川至首尔国际直航航班，与韩国庆山、吉尔吉斯斯坦比什凯克等8个城市结为友好城市，毗邻地区合作发展不断深化，承接国际、国内产业转移成果斐然。五年招商引资到位资金达到1640亿元，引进世界、中国500强企业58家。坚持推进改革创新，完成新一轮政府机构改革，推行“扁平化”管理。行政审批制度改革不断深化，成为西北地区行政审批项目数量最少的城市。建立市民服务中心22个，街道（社区）社会管理和公共服务职能得到加强。自觉接受人大法律监督、政协民主监督和社会监督，共办结人大议案30件、代表建议326件、政协委员提案1851件，办复率均达到100%；提请人大审议通过地方性法规34部，制定政府规章25部，法治政府建设和依法行政水平明显提升。在全国率先出台《党和国家机关及其工作人员不当行为问责办法》等制度，严肃查处“三不”案件，建立重大项目推进、巡视督查机制，政府效能和诚信建设富有成效。引进交通银行、招商银行等各类金融机构10余家，组建铸龙、水务、交通等政府投融资平台，中小企业担保融资机制逐步健全。推进土地流转、集体林权等农村综合改革，农业科技推广服务体系改革成效明显。稳步推进教育、文化、卫生、园林等事业单位人事分配制度改革，自来水、污水等公用事业市场化改革基本完成。审计、仲裁、档案、地方志、红十字等各项工作实现新发展。

各位代表：过去的五年，是银川经济社会发展最快的五年，是城乡面貌变化最大的五年，是人民群众受益最多的五年。发展凝聚汗水，成就来之不易。这得益于自治区党委、政府和市委的坚强领导、科学决策，得益于市人大、市政协的有力监督、鼎力支持，得益于各级干部和全市人民的同心协力、不懈奋斗，还得益于历届政府班子和离退休老同志打下的良好基础。在此，我代表市人民政府，向全市各族人民，向各民主党派、工商联、无党派人士和人民团体，向驻银部队指战员、武警官兵、公安民警，向所有关心支持银川建设发展的社会各界朋友们，表示衷心的感谢和崇高的敬意！

五年的努力奋斗，五年的光辉历程，我们收获了经济社会发展的丰硕成果，积累了实现新跨越的宝贵经验：必须始终坚持科学发展不放松。我们立足市情，发挥比较优势，加快建设“两个最适宜”城市，走出了一条符合银川实际的科学发展之路。今后也必须坚持科学发展，把加快发展、跨越发展作为首要任务，把调整经济结构、转变发展方式作为主攻方向，把保障和改善民生作为根本目的，努力实现率先发展、创新发展、和谐发展、可持续发展。必须始终坚持改革开放不放松。我们坚持深化改革、扩大开放，创新体制机制，破解了一些多年来影响经济发展、社会进步和民生改善的突出问题，发展内生动力和活力不断增强。今后也必须坚定不移地推进改革，坚持对外开放、对内放开，不断释放发展潜力，拓展发展空间，增强发展后劲。必须始终坚持团结稳定不放松。作为少数民族自治区首府，民族团结、宗教和顺、社会和谐是银川最大的优势，也是最亮的城市名片。我们必须切实履行稳定第一责任，巩固发展社会安定团结、和谐稳定的大好局面。必须始终坚持抓好落实不放松。我们把抓落实作为政府的重要职责，确立并大力弘扬“贺兰岿然、长河不息”的银川精神，咬定目标不松劲，拼搏实干不懈怠，狠抓各项工作特别是重大决策、重点项目、重要工作的落实，才取得了今天的发展成就。今后，必须把抓执行、抓落实作为重中之重，加强督查检查和考核奖惩，把各项工作不断向前推进。必须始终坚持自身建设不放松。我们积极转变政府职能，推动管理创新，以政府管理方式转变推动发展方式转变，着力建设规范高效务实清廉的服务型政府。今后也必须不断加强政府自身建设，强化公共服务和社会管理职能，正确处理好政府、市场、社会的关系，让全社会创新创造活力竞相迸发。

在肯定成绩、总结经验的同时，我们也清醒地认识到，目前经济社会发展中还存在不少困难和挑战。一是经济实力还不够强，主导产业规模不大，新兴产业刚刚起步，产业层次有待提升。二是科技创新能力不足，资源能源消耗刚性增长压力较大，城乡发展还不平衡，转变经济发展方式难度增大。三是保障和改善民生的任务艰巨，推进扶贫开发、移民增收的任务尤为繁重。四是干部群众的思想观念、创新意识、开放意识与新形势、新任务要求还有差距。这些问题，我们将在今后工作中采取有力措施，不断加以解决。

二、今后五年的目标任务

各位代表：今后五年，是全面贯彻落实党的十八大精神，率先全面建成小康社会，实现银川跨越式发展的关键时期，也是全面推进宁夏内陆开放型经济试验区和银川综合保税区（以下简称“两区”）建设，深化改革开放，实现银川经济社会转型升级的攻坚时期。党的十八大确定了全面建成小康社会、全面深化改革开放的战略目标，描绘了夺取中国特色社会主义事业新胜利、加快推进现代化、实现中华民族伟大复兴的宏伟蓝图，提出了推进中国特色社会主义“五位一体”的总体布局。特别是明确提出“优先推进西部大开发战略，加大对民族地区、贫困地区扶持力度”，为我市科学发展、跨越发展带来了新机遇，提供了新动力。自治区第十一次党代会提出了建设和谐富裕新宁夏、与全国同步进入全面小康社会的奋斗目标，要求银川市在“四个方面继续走在全区前列”、发挥“三个方面龙头作用”，打造“宁夏航母”和“中国迪拜”，为我市科学发展、跨越发展指明了新方向，提出了新要求。市第十三次党代会提出了“四个新跨越”的奋斗目标，市委、政府确立了“2258”工作思路和任务，为我市科学发展、跨越发展明确了新目标，描绘了新蓝图。

面对党中央、自治区党委、政府和市委提出的新要求，面对全市人民过上更好生活的新期待，我们必须牢固树立强烈的发展意识、责任意识和忧患意识，紧紧抓住新一轮经济发展的机遇、新一轮扩大开放的机遇、新一轮城镇化的机遇、新一轮社会建设的机遇、新一轮生态文明建设的机遇，解放思想，凝心聚力，真抓实干，改革创新，把重大战略机遇期转变为推动银川跨越式发展的黄金发展期，共同创造更加美好的明天！

今后五年政府工作的总体思路是：深入贯彻落实党的十八大及自治区第十一次党代会、市十三次党代会精神，坚持以邓小平理论、“三个代表”重要思想、科学发展观为指导，紧紧围绕实施“两大战略”和推进“两区”建设，全面落实“2258”工作思路和任务，统筹推进工业化、信息化、城镇化和农业现代化，更加注重提高经济发展质量、效益，更加注重深化改革、扩大开放，更加注重创新驱动发展，更加注重保障和改善民生，更加注重维护社会和谐稳定，打造现代化、国际化、生态化城市，率先全面建成小康社会，建成“两个最适宜”城市，建设实力倍增、文明开放、城市靓丽、生态优美、民族团结、人民幸福的和谐富裕新银川。

主要预期目标是：在全面完成市十三次党代会“四个新跨越”目标基础上，努力实现新的突破。到2017年，经济实力大幅提升，实现地区生产总值、地方公共财政预算收入、全社会固定资产投资比2012年翻一番。发展方式明显转变，优势特色产业规模明显扩大，效益显著提升，现代服务业比重大幅提高；高新技术产业增加值占规模以上工业增加值比重达到30%以上；科技进步对经济增长的贡献率达到55%以上，进入国家创新型城市行列；“资源节约型、环境友好型”社会建设取得重大进展。内陆开放新格局基本形成，对内对外开放实现战略新转变，打造向西开放的大窗口、聚集产业和金融的大平台。城乡一体化全面推进，城镇化进程全面加速，质量明显提升，城镇化率达到80%以上；“新五创”目标全面实现，建成现代化区域性中心城市。人民生活水平全面提高，基本公共服务均等化初步实现，教育、卫生、文化等社会事业发展指标高于全国平均水平，在周边毗邻地区领先地位牢固确立；城乡居民人均收入比2012年翻一番，社会保障水平进一步提高。

围绕上述目标任务，重点在“六个方面”实现新突破：

（一）加快建立现代产业体系，在建设实力银川上实现新突破。壮大经济实力、提升经济竞争力是银川率先发展、领先发展的根本路径。要立足资源优势和产业实际，以发展高端产业和产业高端为重点，建立以战略性新兴产业为先导、以先进制造业和现代农业为支撑、以现代服务业和总部经济为核心的新型产业体系，显著提升经济竞争力。深入推进工业振兴工程，全面加快新型工业化进程。加快培育发展战略性新兴产业，着力打造新能源、新材料、装备制造及再制造、高新技术等产业集群；大力推进优势特色产业转型升级，加快技术创新和“两化”深度融合，促进产业链向高附加值延伸。力争到2017年规模以上工业增加值突破1000亿元大关，比2012年增长1.5倍以上。深入推进现代农业示范工程，大力发展“三精农业”。坚持园区化、标准化、生态化、规模化方向，加快农业科技创新，促进优势特色产业集约发展，打造一批优质农产品产业带、一批加工销售龙头企业和一批知名品牌，建成全国现代农业示范区和农产品安全区。深入推进服务业提升工程，促进服务业发展提速、规模扩大、结构优化。把阅海湾中央商务区打造成高端服务业、总部经济和会展经济高地，建成区域性现代服务业中心。实施金融强市战略，建成区域性金融中心；大力发展现代物流业，建成全国重要的区域性物流中心；促进商贸服务业等传统产业优化升级，建成区域性商贸中心；打造全国知名的旅游度假休闲目的地城市，力争把文化旅游产业打造成我市支柱产业。深入实施项目带动战略，着力引进大企业、建设大项目、培育大园区，确保全社会固定资产投资持续较快增长，为保增长、转方式、调结构提供有力支撑。

（二）加速推进城镇化，在建设宜居银川上实现新突破。宜居宜业是银川的优势和潜力所在。要围绕打造“两个最适宜”城市，着力推进城乡规划、基础设施、产业发展、公共服务一体化，加快形成工业化与城镇化良性互动、城镇化与农业现代化相互协调的城乡统筹发展新格局。建立“全域银川”规划体系，完善重点区域控制性详规、村镇体系规划、综合交通规划，形成相互衔接、覆盖城乡的规划体系。编制《产城一体规划》，调整产业战略功能分区，形成“一区一主业”发展格局，构建新型产城关系。坚持“提升主城、建设新城、重心东扩、跨河发展”，优化城市整体格局，提升城市承载功能。以改善旧城区交通环境、提高宜居水平为目标，坚持系统规划、稳步开展，突出提质限容、做好“减法”，全面完成旧城更新改造，使历史文化名城特色更加鲜明。高起点规划、高标准建设、高水平发展滨河新区，初步建成产业功能区、文化旅游休闲区、城市生活服务区“三大功能区”，打造城市建设新亮点、经济发展新引擎和黄河金岸璀璨明珠，向国家级新区迈进。推进灵武、永宁、贺兰县城扩容提质，加快“同城化”步伐，大力发展特色小城镇，合理确定村庄规模，引导农村居民向小城镇和中心村集中居住，打造城乡融合、多极支撑、组团发展的现代化城市圈。加快推进户籍管理及配套制度改革，有序推进农村人口市民化，努力实现城镇基本公共服务全覆盖。加快以综合交通体系为重点的基础设施建设，完善延伸城乡路网；完成兵沟、永灵等跨黄河大桥及连接线、铁路运输中心等交通枢纽建设；推动包兰铁路扩能改造，争取开通银川至西安等城市高速列车；加快河东机场三期扩建，开通与国内更多城市的直达航线以及到东北亚国家的国际航线，形成快捷通畅的现代化立体交通网络。完善城乡供水、供气、供电及防洪防汛设施。实施《“智慧银川”建设与发展十年规划》，加快资源整合和集成共享，建设“智慧银川”。

（三）打造“两区”核心区，在建设开放银川上实现新突破。对外开放是银川走向国际化、现代化的必由之路，“两区”建设是银川跨越式发展和对外开放的重大机遇。要大力实施开放带动战略，以内陆开放型经济试验区为引领，以阅海湾中央商务区、综合保税区、滨河新区为载体，先行先试、敢闯敢试、创新突破，加快构建全方位对外开放格局。以阅海湾中央商务区为核心，规划建设阅海湾经济区，打造向西开放与国际交流先导平台、现代化国际化城市核心展示平台、都市生态与低碳经济示范平台。推进向东北亚开放，扩大与韩国、俄罗斯等国家在园区建设、医疗卫生、文化旅游等方面的合作。以综合保税区为先导，引领外向型经济快速发展，打造内陆开放型经济试验区的核心和引擎。完善产业配套区和附属区功能，发展保税物流、保税加工、保税服务和口岸作业业务，建设全国重要的羊绒制品等优势特色产业出口加工区和集聚区。以滨河新区为载体，推进区域经济合作，打造承接国内外产业转移示范区。全面深化与珠三角、长三角、环渤海、成渝经济圈等区域的交流与合作，高起点承接产业转移和产业链的跨区域延伸；加强与陕甘蒙等毗邻地区、能源“金三角”、呼包银经济圈的战略协作，提升区域中心城市的聚集辐射带动能力。

（四）积极构建“两型社会”，在建设美丽银川上实现新突破。“塞上湖城”是银川最值得珍惜的靓丽名片。要牢固树立生态文明理念，加快建设“资源节约型、环境友好型”社会，促进资源环境与经济社会全面协调可持续发展。深入实施主体功能区规划，严格按照功能定位发展。加强节能减排目标管理和考核评价，抓好工业、建筑、交通和公共机构等重点行业、重点用能企业节能监管，完善落后产能淘汰机制。大力发展循环经济，全面推行清洁生产，提升资源综合利用和污染防治水平。坚持集约节约用地，清理处置闲置土地，严格水源地保护，提高中水利用率，农村环境连片整治实现全覆盖。加快创建国家生态园林城市，实施城市水系连通工程，加强湖泊湿地生态修复、保护和利用，全力打造“塞上湖城、西北水乡”。建设“黄河外滩”绿色景观线、阅海环湖和唐徕公园生态休闲线、贺兰山东麓及银西绿色防护线。打造城市景观绿化精品，建成“绿博园”，申办2015年第三届中国绿化博览会。到2017年，全市森林覆盖率达到20%以上，为建设国家西部生态安全屏障做出应有贡献。

（五）大力改善民生，在建设幸福银川上实现新突破。提升人民群众幸福指数是政府工作的永恒主题。要坚持民生导向、富民优先，加快健全基本公共服务体系，让全市人民享受更多经济社会发展成果。实施就业优先战略，深入推进全民创业带动就业，城镇新增就业15万人。实施城乡居民收入“倍增计划”，稳步提高企业最低工资标准和城乡低保标准，着力提高中低收入者收入，创造条件增加群众的经营性收入、财产性收入，缩小城乡、行业和区域之间收入差距。健全完善覆盖城乡居民的社会保障和社会救助体系，大力开展慈善城市创建活动。建设保障性住房4.1万套，实现应保尽保。加快实施扶贫攻坚计划，全面完成自治区下达的7.8万人的生态移民安置任务。坚持教育优先发展，加大中小学、幼儿园建设投入，逐步提高教师待遇。完善教育考核评价机制，大力实施素质教育和特色化办学，着力提高教育质量。推行集团化办学，促进民办教育规范发展。建设国家健康城市和食品安全城市，完善基层卫生服务体系，加快推进公立医院改革，健全食品药品安全监管体制机制，实现人人享有基本医疗卫生服务目标。完善四级公共文化服务体系，扩大文明城市成果，全面提升市民素质和城市文明水平。

（六）创新社会管理，在建设和谐银川上实现新突破。和谐包容是银川最优秀的人文品质。要进一步优化环境、创新服务，最大限度地促进社会和谐。改进政府公共服务和社会管理方式，完善市、县（市）区、乡镇（街道）、村（社区）四级政务服务体系，加强基层社会管理和服务体系建设。健全重大决策社会稳定风险评估机制，完善矛盾纠纷排查调处机制，加强专业调解队伍建设，拓宽畅通群众诉求表达、利益协调、权益保障渠道。推进“诚信银川”建设，建立完善全市统一的征信体系和失信惩戒机制。深化“平安银川”创建，完善立体化社会治安综合防控体系，严格安全生产监管，加强防灾减灾体系建设，提高公共安全和突发事件应急处置能力。创建全国双拥模范城“八连冠”，提高国防后备力量建设质量。争创民族团结进步模范城市，加强宗教事务规范化管理，切实维护民族和睦、宗教和顺、社会和谐的大好局面。

三、2013年主要工作

各位代表：2013年是全面贯彻落实党的十八大精神的开局之年，是实施“十二五”规划承前启后的关键之年，是为全面建成小康社会奠定坚实基础的重要一年。做好明年的工作，对于新一届政府开好局、起好步，加快建设“两个最适宜”城市，推动跨越式发展，具有重大意义。

政府工作总的要求是：认真贯彻中央经济工作会议精神，抢抓“两区”建设重大机遇，紧紧围绕建设和谐富裕新宁夏和“两个最适宜”城市的目标，以科学发展为主题，以经济工作项目化为主线，深入实施“2258”工作思路，稳中求快，开拓创新，扎实开局，着力推动产业优化升级，着力统筹城乡发展，着力深化改革开放，着力保障和改善民生，着力维护社会和谐稳定，着力加强政府自身建设，奋力开创银川科学发展、跨越发展新局面。

主要预期目标是：地区生产总值增长13%以上，地方公共财政预算收入增长15%以上，全社会固定资产投资增长25%以上，社会消费品零售总额增长18%以上，外贸进出口总额增长15%，城镇居民人均可支配收入和农村居民人均纯收入增长13%以上，居民消费价格总水平涨幅控制在4%以内，城镇登记失业率控制在4%以内，单位地区生产总值能耗、主要污染物减排完成自治区下达的控制指标。

重点抓好以下九个方面工作：

（一）全力建设“两个新区”，构建跨越发展大格局

加快阅海湾经济区开发建设。完成规划设计，完善优化功能布局，基本建成中央商务区道路、绿化、水系等基础设施。实施“510”工程，抓好绿地中心、新华联广场、鸿曦铂金酒店等项目，开工建设银川国际交流中心和规划展示馆，引进世茂集团、远大集团等大企业，加快建设国际化、现代化、生态化城市新区。

全面推进滨河新区建设。完成总体规划和专项规划，推进横城村等重点片区拆迁和土地整理，启动兵沟、红墩子黄河公路大桥及连接线工程，加快给排水、污水处理等基础设施建设。抓好黄河银川段航道疏浚及堤岸砌护整治，完成“银川舰回家”工程，建设军事主题公园。实施国际医疗中心、教育基地、移民安置区等项目建设，力争滨河新区建设集中突破。

（二）加快“五大园区”建设，力促工业转型升级

推进园区扩容提质。延伸宁东能源化工基地产业链，加快建设滨河新区工业园，启动潮商工业园、台商产业园、韩国中小企业园建设。突出抓好银川经济技术开发区建设，加快培育发展战略性新兴产业和大型龙头企业，提升可持续发展能力。开工建设生态纺织产业示范园基础设施和核心项目，建设灵武园区。推动银川高新技术产业开发区建设，大力发展羊绒精深加工，壮大产业集群，引领经济结构调整和发展方式转变。高标准规划建设银川科技园，建设研发中心和科技人才大厦，推进区市共建，合作建设宁东科技园。推进工业向园区集中，严格限制在工业园区外新建工业企业，鼓励市内现有工业企业搬迁入园。建立园区评价考核机制，切实提高投资强度和产出效益。

提升工业发展水平。促进能源化工产业优化发展，大力发展新型煤化工项目及产业链延伸产品。开工建设石油天然气化工产业园。引导电工电气企业兼并重组和管理创新，培育发展竞争力强、带动力大的龙头企业。改造提升特色农产品加工等传统产业，实施80个技改项目，加强品牌建设。大力发展新能源、新材料、先进装备制造等新兴产业，重点扶持银和新能源、隆基硅业、巨能机器人等自主创新能力强、高成长性企业做大做强。大力发展信息产业，加快培育葡萄酿酒、家具装饰等新的经济增长点。落实支持小微企业发展的政策措施，建立大型龙头企业与中小微企业协作配套机制，培育一批“专精特新”中小微企业。全年工业投资增长20%以上，规模以上工业增加值增长16%以上。

打好节能减排攻坚战。推进管理、技术、结构节能，加强重点用能企业能源审计，推行合同能源管理。加大环境监察执法力度，严厉打击违法违规排放行为。启动东部热电配套热力管网改造工程，逐步拆除市区燃煤锅炉，监测公布PM2.5等指标。严格执行建筑节能标准，推进绿色建筑试点。继续实施节能产品惠民工程，推动节能环保产业发展。

（三）突出提质增效，推动现代农业上水平

加快现代农业示范园区建设。扩大设施园艺、奶产业、有机大米、“适水”产业、酿酒葡萄等优势产业规模，建设一批标准化和产业化程度较高的生产基地，发挥新品种试验展示、新技术集成推广、新模式示范引领作用。新建5个示范园区，改造提升10个老旧园区，新增设施园艺2万亩、有机水稻4万亩、酿酒葡萄1万亩，奶牛存栏达到17万头，全年农业总产值增长5%以上。

培育壮大龙头企业。着力培育一批规模型、创新型和成长型农业产业化龙头企业，重点扶持20个龙头企业做强做优。完善农产品营销体系，加快推进农超对接、农产品直销，积极发展连锁经营、冷链体系等新型流通方式。狠抓品牌创建，加强农产品质量监管，完成10个以上绿色、有机农产品认证认定，促进农产品加工转化和增值增效。

建立新型农业经营方式。加快农村土地、房屋确权登记，建立产权交易平台，引导土地经营权合法有序流转，推进土地规模化、集约化经营。大力发展农民专业合作组织，创建市级以上示范社36个，提高农民组织化程度。完善农业科技服务体系，加大新型农民培训力度。

加强农村基础设施建设。大力开展农田水利建设，实施沟渠塘田林路综合整治，建设巩固高标准农田50万亩，改造中低产田30万亩，新增高效节水灌溉5万亩，新建农村道路200公里，保持好塞上江南田园风光。加快推进掌政、镇北堡等特色小城镇建设，加强农村住宅建设管理，推广农村“社区化”，新建“塞上农民新居”示范点9个，明显改善农村生产生活条件。

（四）大力推进“两带”建设，加速现代服务业发展

打造文化旅游核心品牌。加快东西两条文化旅游带建设步伐，全力推动滨河新区5A级景区规划建设，推进华夏河图、希诺国际健康城、贺兰山东麓葡萄文化博览中心等项目，抓好西夏陵申遗及西夏博物馆迁建工程。推进与首旅、首汽等集团的合作，启动建设汽车摩托车旅游运动基地和自驾游基地。全方位扩大营销宣传，全年旅游接待人数和旅游总收入分别增长20%和22%以上。

提升现代服务业发展水平。出台《关于加快促进区域性物流中心建设发展的若干政策》，加快推进陆港、国际公铁物流城建设，开工建设新百现代物流和润恒农副产品物流产业园，培育现代物流企业，构建“大物流”发展格局。优化商业布局，积极引进国内外知名商贸企业，构建金凤区大世界广场－万达广场－阅海新天地新商圈，增强商业集聚功能。加快发展生产型服务业，大力发展现代服务业和总部经济、会展经济，办好第六届银川（国际）汽车博览会等大型展会。扎实推进电子商务示范城市建设，加快培育文化创意、软件动漫、服务外包等新兴业态。

（五）强力实施“四大工程”，全面提升城市功能品位

实施旧城更新改造工程。坚持政府主导、项目带动、市场运作、多方参与，充分尊重群众意愿，实施三区重点片区老旧小区改造，加快改造城中村、城边村，完善旧城区公共服务和市政基础设施，大幅增加绿地、停车场，提高亮化美化水平，改善市民居住环境。

实施道路畅通工程。加快完善城市路网，新建续建友爱中心路连接线、六盘山路等16条道路，改造延伸西桥巷、鼓楼北街等10条街巷，实施新华西街、湖滨西街跨唐徕渠桥及道路打通工程。优先发展公共交通，完善公交线网布局和站点设置，优化快速公交一号线运营组织，启动综合交通枢纽项目建设，创建国家“公交都市”示范城市。规划建设城市公共自行车系统，新建公共自行车亭300个，倡导低碳绿色环保出行。加强交通智能化管理，优化交通组织和信号灯配时，抓好节点改造，有效缓解市区交通拥堵。

实施生态品位提升工程。加快推进湖泊湿地连通，完成爱伊河北京路码头至北塔湖水系景观一期工程，启动城市南部水系建设，实施七子连湖扩整工程。完善中山公园等城市公园综合功能，加快旧城区绿地精品化改造。推进唐徕渠六期整治和“绿博园”建设，完成贺兰山路等7条城市主干道生态景观林带及道路配套绿化工程。抓好黄河金岸银川段生态景观提升、滨河新区大环境绿化和阅海环湖景观绿地改造，大力推动全民植绿、爱绿护绿，全年新建完善园林绿化面积500公顷，造林6667公顷。

实施“洁净银川”深化工程。强化城乡环境卫生综合整治，抓好城市出入口、城乡结合部、主要道路沿线整治，坚决清理占道经营和马路市场，规范早市夜市管理，加强“三乱”治理，从严整治建筑垃圾清运撒漏等突出问题。抓好特色街区改造，规范门头牌匾、户外广告、建筑外立面装饰。建立建筑垃圾和农村生活垃圾收集转运处理机制，统筹城乡环卫工作。加快建立“大城管”体制机制，加强“数字城管”系统应用，推进城市全方位、精细化管理。积极开展爱国卫生运动，严格落实“门前三包”责任制，让我们的城市更干净、更整洁、更美丽。

（六）抓紧抓好银川综合保税区建设，促进外向型经济快速发展

加快综合保税区规划建设。高起点编制完成综保区及配套区规划，实现控制性详细规划与滨河新区、临空经济区、河东机场三期等规划无缝对接。出台综保区管理办法及有关政策。加快建设海关检验检疫查验设施、物流仓储、对外交通等基础设施，同步引进外向型出口加工企业和项目，确保2013年9月前建成通过验收并封关运行。

扩大对阿对东北亚开放合作。举办中韩（银川）活动周，稳定银川至首尔国际航班运营，组建股份制航空公司，开展“第五航权”试点，争取“离银免税”和“落地签”政策。提高招商引资质量和实效，突出招大引强、招研引智，积极引进大项目和战略投资者，全年招商引资实际到位资金增长25%以上。出台《加快外经贸发展的实施意见》，大力发展外向型经济。

（七）推进改革创新，增强发展内生动力

推进重点领域改革。进一步明确市、辖区两级事权和财权，建立财政一般性转移支付制度和稳定增长机制。集中财力办大事，加大对主导产业、重点项目、基本公共服务的财政支持，在社会事业和社会管理领域更多采取购买服务、民办公助等方式，放大财政资金效益。建立向上向外争项目、争资金考核奖励机制。建立完善政府投融资平台市场化稳定运行机制，组建科技、住房等投资公司。推行国有资本经营预算制度，制定促进非公经济发展的政策措施，放宽民间资本投资领域，降低准入门槛。积极稳妥推进事业单位分类改革。

推进创新型银川建设。坚持创新引领发展，加大科技投入，加快科技创新体系建设。围绕重点产业和领域，突出企业主体作用，推动产学研结合，实施共性关键技术研发和科技成果转化项目100个。提升与中科院、浙江大学等国家级科研机构、高等院校合作水平，搭建科技服务平台，建设科技成果转移转化中心和中试基地。新培育高新技术企业10家，国家级、自治区级企业技术中心4家，专利申请量和授权量增长15%以上，增强科技创新能力。

推进“人才特区”建设。大力实施 “引凤”工程，面向全国引进一批创新型和外向型人才，加大柔性引才力度。深入推进“育英”工程，实施以专业技术人才、高技能人才和农村实用人才为重点的人才培养计划。优化人才发展环境，提高人才待遇，健全完善以实绩为导向的人才评价考核和激励保障机制，让各类人才人尽其才，才尽其用，大显身手。

（八）坚持民生为重，全面加强社会建设

倾力保障和改善民生。继续为民办好10件实事。建成市人力资源市场，加强公共就业服务和职业技能培训，发挥好政府投资项目带动就业的作用，着力解决高校毕业生、农民工、就业困难人员和退役军人就业问题，城镇新增就业5万人，农村劳动力转移10万人。巩固创业先进城市创建成果，完善创业扶持政策，健全服务体系，实施就业创业培训1万人以上，提升创业示范园区（基地）孵化功能，全年培育小企业1000个、小老板1000个。推进社会保障扩面提标，加快统筹城乡居民养老保险和医疗保险。鼓励引导社会力量兴办养老服务业，建设10个老年人日间照料中心。新建各类保障性住房40万平方米，建立完善分配、管理和退出机制，让住房困难家庭真正受益。促进房地产业健康稳定发展。加强居民生活必需品价格监测调控，新建改造标准化蔬菜生产基地10个、菜市场10个，规范社区蔬菜直销店运营管理，及时启动物价补贴联动机制。扎实推进蔬菜肉品流通追溯体系建设试点。统筹推进扶贫攻坚和生态移民工程，加强培训转移、产业扶持和公共服务，完成4000户生态移民安置任务，移民地区农民人均纯收入达到6000元，增长20%以上。

加快发展社会事业。实施优质教育扩面提升工程，采取名校带动、校际联盟、结对帮扶等方式，促进优质教育资源跨区域整合，新建改造中小学16所、幼儿园14所。推进义务教育均衡发展，支持各县（市）区率先实现义务教育均衡发展目标。鼓励高中教育特色化、多样化发展，创新发展职业教育，加快宁夏幼儿师范学校“升格”。深入推进公立医院改革，实施中俄韩医疗合作项目和口腔医院迁建工程，推进社区卫生服务机构和村卫生室标准化建设，提高基层卫生服务水平。大力推进全民健身工程，深化创建“幸福家庭”活动，促进人口计生公共服务转型发展。加快创建国家公共文化服务体系示范区，实施市文化艺术馆建设和图书馆数字化改造，完成三区“两馆”建设，提升村级（社区）文化活动室功能，推进“踏歌起舞、幸福银川”文化工程。实现农村直播卫星户户通全覆盖。

提高社会管理科学化水平。建设社会管理创新综合平台，全面推进社区“网格化”管理，积极引导社会组织和民间团体参与社会管理服务，提高社区工作者待遇。进一步完善“三调联动”机制，深入开展矛盾纠纷大排查、大调处，强化信访维稳工作领导责任。建立“以房管人、以证管人、以业管人”的流动人口服务管理新模式，加强社区矫正和刑释解教人员服务管理工作。深入创建“平安银川”，扎实推进“六五”普法，扩大法律援助覆盖面。强化安全生产监管责任和企业主体责任落实，加强道路交通、消防、建筑施工、危险化学品等重点领域安全隐患排查整治，切实增强群众安全感。创新推进民族团结进步、和谐寺观教堂创建活动。深入开展国防教育，加强国防动员和民兵预备役建设。

（九）着力加强自身建设，打造为民高效务实廉洁的服务型政府

做到依法行政。加强政府立法和制度建设，规范行政执法行为，加强行政复议工作，促进严格公正文明执法。大力推进政务公开，推行“电视问政”等创新做法。完善重大事项专家论证、公众参与、合法性审查、集体决策等制度，推进决策科学化、民主化、法治化。主动接受市人大及其常委会和人民政协的监督，不断提高人大议案、政协提案办理质量，认真听取各民主党派、工商联、无党派人士和人民团体的意见，支持新闻舆论监督和社会监督，把政府工作置于广泛的监督之下。

做到高效施政。建设市民中心。完善行政审批运行机制，推进“简政放权”和“三减两提高”，推行重大项目“绿色通道”和“全程代办制”，促进审批办事效率再提速。推行政府绩效管理，完善重点工作公开承诺、目标管理和督查考核机制。强化行政问责，坚决治理公务人员“庸懒散软奢”等不当行为，建设讲学习、重实干、高素质公务员队伍，提高政府效能和执行力。推进营造风清气正发展环境活动常态化、长效化，着力解决企业和群众反映强烈的突出问题，为民营经济和中小企业发展创造良好条件，打造西北一流的投资创业环境。

做到廉洁从政。认真落实党风廉政建设责任制，加强政府内部监督、行政监察和审计监督，突出抓好工程建设、土地出让、征地拆迁、政府采购等重要领域和关键环节的监督。贯彻落实中央八项规定，大力改进工作作风，从政府领导班子成员做起，带头深入基层联系群众、调查研究、解决实际问题；带头开短会、讲短话、发短文、集中精力抓落实。推进财政预算、决算公开，厉行勤俭节约。严肃查处违纪违法案件，切实纠正各类损害群众利益的不正之风，以政府勤政廉政新成效取信于民。

各位代表！

肩负新使命，我们的责任重大而光荣；展望新蓝图，银川的前景光明而美好。让我们在自治区党委、政府和市委的坚强领导下，深入学习贯彻十八大精神，大力践行“贺兰岿然、长河不息”的银川精神，坚定信心，凝聚力量，攻坚克难，加快银川跨越式发展，为建设和谐富裕新宁夏和“两个最适宜”城市，率先全面建成小康社会而努力奋斗！
